# High-performance visible light photodetectors based on inorganic CZT and InCZT single crystals

**DOI:** 10.1038/s41598-019-48621-3

**Published:** 2019-08-27

**Authors:** Mohd. Shkir, Mohd Taukeer Khan, I. M. Ashraf, Abdullah Almohammedi, E. Dieguez, S. AlFaify

**Affiliations:** 1 Advanced Functional Materials and Optoelectronics Laboratory (AFMOL), Department of Physics, College of Science, King Khalid University, P.O. Box 9004, Abha, 61413 Saudi Arabia; 2 Department of Physics, Faculty of Science, Islamic University of Madinah, Madinah, 42351 Saudi Arabia; 3 Department of Physics, Faculty of Science, Aswan University, Aswan, 81511 Egypt; 4 Crystal Growth Lab, Departamento de Física de Materiales, Universidad Autónoma de Madrid, Madrid, 28049 Spain

**Keywords:** Electronic devices, Electronic properties and materials

## Abstract

Herein, the optoelectrical investigation of cadmium zinc telluride (CZT) and indium (In) doped CZT (InCZT) single crystals-based photodetectors have been demonstrated. The grown crystals were configured into photodetector devices and recorded the current-voltage (*I-V*) and current-time (*I-t*) characteristics under different illumination intensities. It has been observed that the photocurrent generation mechanism in both photodetector devices is dominantly driven by a photogating effect. The CZT photodetector exhibits stable and reversible device performances to 632 nm light, including a promotable responsivity of 0.38 AW^−1^, a high photoswitch ratio of 152, specific detectivity of 6.30 × 10^11^ Jones, and fast switching time (rise time of 210 ms and decay time of 150 ms). When doped with In, the responsivity of device increases to 0.50 AW^−1^, photoswitch ratio decrease to 10, specific detectivity decrease to 1.80 × 10^11^ Jones, rise time decrease to 140 ms and decay time increase to 200 ms. Moreover, these devices show a very high external quantum efficiency of 200% for CZT and 250% for InCZT. These results demonstrate that the CZT based crystals have great potential for visible light photodetector applications.

## Introduction

Cadmium zinc telluride (CdZnTe or CZT), a p-type semiconductor have recently gained much attention in the key detector technologies. The main attractive aspect of CZT crystals are (i) wide band gap (~1.68 eV) which is necessary for room temperature operation, (ii) a large photon absorption cross section (∼10^4^ cm^2^/g for 1 keV photon energy; for photon energy <50 keV the absorption efficiency is >95%) for efficient conversion of optical energy in to electrical energy, and (iii) high resistivity (10^10^ Ω.cm) to minimize the noise due to limiting the leakage current^1–5^. These specific properties allow its applications in a wide range of devices such as X-ray or γ-ray detector, nuclear spectroscopy, medical imaging, radiation sensors, photorefractive, etc.^6–10^. Due to such venerable applications of CZT crystals in detector technology, it has already started to replace the existing X-ray and γ-ray detectors as well as other semiconductors detectors.

However, there are few drawbacks associated with CZT crystals. Firstly, it has relatively high concentrations of impurities and inherent defects that cause small drift length^11,12^. Secondly, low hole mobility, which caused by the trapping of charge carries^13^. These downside causes the poor device performance and hence limiting the applications of CZT crystals. Therefore it is essential to compensate these impurities and defects with the introduction of additional donor impurity into the CZT crystals. The additional donor impurity accomplishes a balance between electron and hole concentration and compensate the native defects presents into the CZT crystals^14^. Indium is considered one of the most promising dopants for CZT crystals in a doping concentration range of 1 × 10^19^ at./cm^3^ to 8 × 10^19^ at./cm^3^ ^15^.

Recently, silicon and germanium based photodetectors have been widely studied^16–19^, but their relatively low external quantum efficiency (EQE), poor responsivity, high dark currents and long response time limit the commercialization of these devices. Most recently, photodetectors based on lead dihalide (LDH)and transition metal dichalcogenides (TMD) have shown promising results but they suffer from environmental instability due to oxygen chemisorption effect^20–24^. Photodetectors based on PbI_2_ and PbFI, reported by Tan *et al*. showed higher photocurrent in a vacuum than those in air^25^. Similar results were also observed for ternary Ta_2_NiSe_5_ based photodetector where photocurrent in vacuum was larger than that in ambient^26^. Huo and co-workers observed responsivity of 18.8 m A W^−1^ in vacuum for the tungsten disulfide multilayer based photodetector, which is drastically reduced in the air to 0.2 μA W^−1^ ^27^. Another study on molybdenum disulfide based phototransistor showed a maximum responsivity of 2200 A W^−1^ in a vacuum, which was reduced to 780 A W^−1^ in air^28^. These studies urges to find an alternate material for a photodetector which is highly efficient as well as have good environmental stability. As discussed above, the CZT owing good optoelectrical properties for radiation detectors, as well as have good environmental stability. Therefore, it is expected that it can also deliver good performance in visible photodetector technology.

To the best of our knowledge, no systematic study on CZT and InCZT based photodetector have been reported so far. In the present work, we have grown the pure and indium doped CZT single crystals and demonstrates the photocurrent generation mechanism in photodetectors based on these crystals. For this, we have measured the illumination dependence current-voltage (*I-V*) and current-time (*I-t*) curves and calculated the detectivity, responsivity, EQE, photoconductivity and switching time of photodetectors.

## Experimental Details

**Figure 1:**
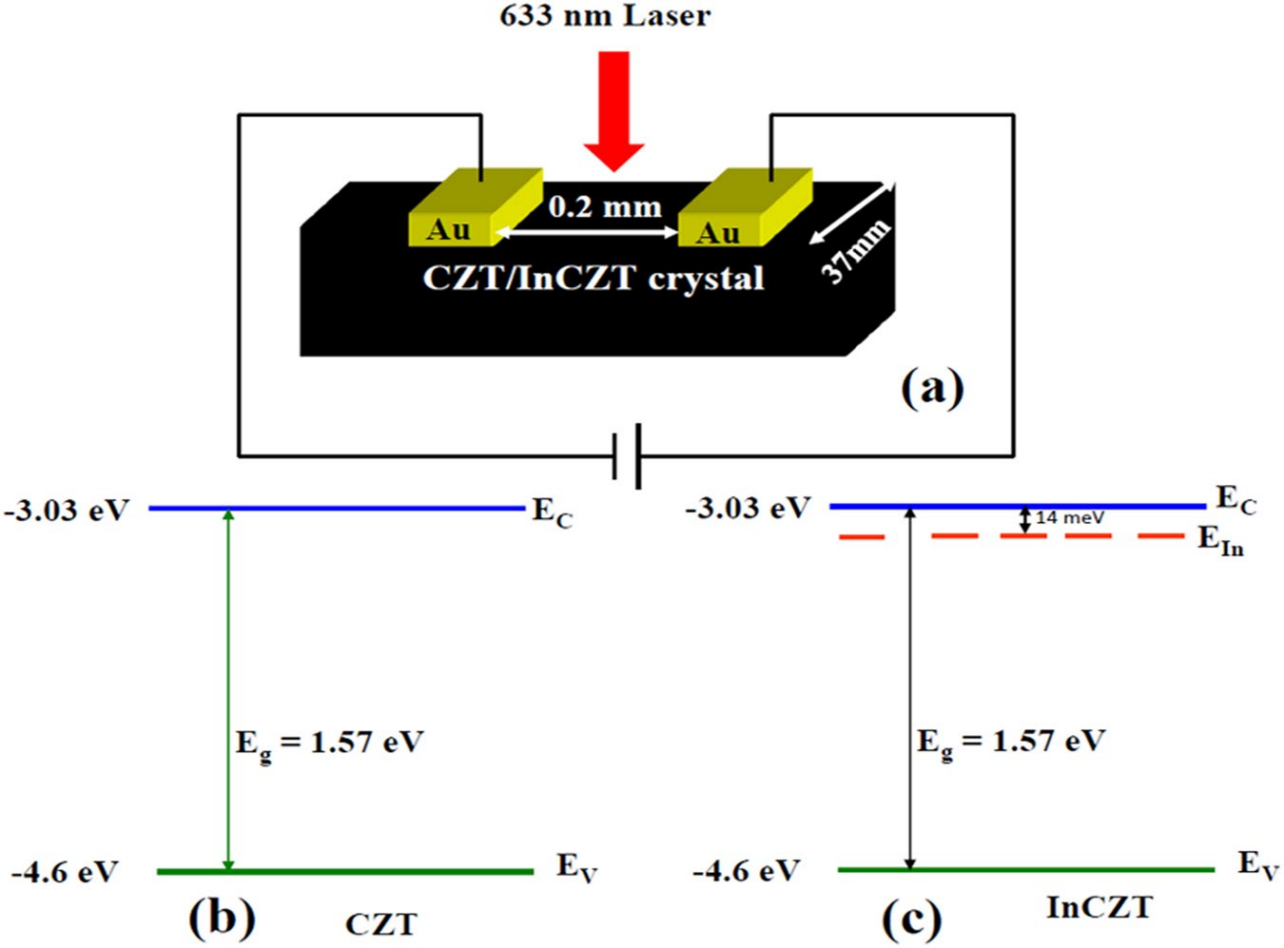
(**a**) Schematic diagram of fabricated photodetector device from grown crystals and schematic of the energy levels of (**b**) CZT and (**c**) InCZT. Here, E_C_ and E_V_ stands for conduction band edge and valance band edge, respectively, and E_In_ is the donor level due to In doping in CZT crystal.

The cut and polished rectangular specimens from grown crystals^6,12^ were prepared and subjected to X-ray diffractometer (XRD) to confirm the growth direction. A typical photodetector consists of a semiconductor as a channel, with two ohmic contacts affixed to opposite ends of the channel as depicted in Fig. 1(a). For the fabrication of devices, a pair of Au electrodes (~20 nm thick) with 200 μm apart was evaporated by the sputtering unit on the grown rectangular crystals. These electrodes serve as source and drain electrodes for the photodetector device. Gold has a work function of ∼5.0 eV, closely match to the work function of CZT (4.6 eV), which make it a good choice for the injection/collection of charge carriers at the source/drain electrodes^29^. The performance of the fabricated photodetector devices was analyzed in an optical cryostat (Oxford optistat DN_2_) under the evacuation of ~10^−4^ Torr. Programmable digital electrometer (Keithley 6517b) along with an ammeter was used to measure the *I-V* characteristics of devices. A tungsten lamp linked with a power variance was used to attain the preferred illumination. The whole experimental display is exposed in scheme-I (see Supplementary Data). The effect of light intensity on transient photoconductivity was studied by using the previous circuit shown in scheme-I (see Supplementary Data). The photocurrent as a function of time was measured using the programmable digital electrometer interface with software package 6517 Hi-R Step Response. The rise and decay photocurrent were extracted by subtracting the dark current from the total measured current. The illumination and darkness time were controlled via SR540 digital chopper controller. Hall effect measurement at 300 K was studied on samples by applying the voltage of 10 Volt under the magnetic field of 3700 gauss. The value of the carrier concentration was determined by calculating the value of the Hall coefficient. Hall mobility was calculated by using the values of electrical conductivity and Hall coefficient^30^. The carrier life time was calculated by transient photoconductivity measurements as reported in previous paper^31^.

## Results and Discussion

**Figure 2:**
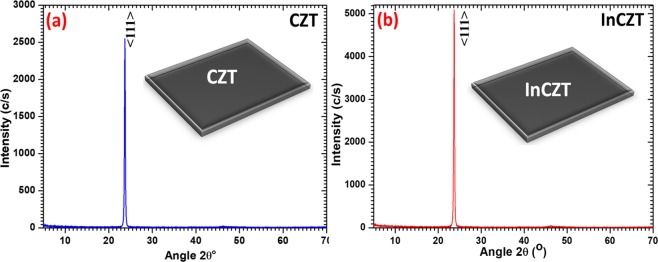
XRD pattern of (**a**) CZT and (**b**) InCZT crystal specimens.

XRD patterns of CZT and InCZT rectangular crystals specimens (inset of figures) are displayed in Fig. 2(a,b), respectively. Both patterns display only a single diffraction peak observed at an angle ~24.463° (d = 3.63580 Å) for CZT and 24.230° (d = 3.67021) for InCZT. The observed XRD peaks in both specimens correspond to the cubic crystal system of space group F-43mE (216) and crystals were grown in the direction of (111) plane, confirmed from standard JCPDS# 50–1438.

The energy band diagram for both CZT and InCZT crystals are shown in Fig. 1(b,c), respectively. The band gap of $C{d}_{1-x}Z{n}_{x}Te$ (CZT) crystal vary from 1.53 eV to 1.65 eV depending upon the value of x according to the relation^8^:

1 $${E}_{g}=1.510+0.606x+0.139{x}^{2}eV$$

With x = 0.1 in the present work, the band gap of CZT was calculated to be ∼1.572 eV. Takahashi and Watanabe have reported the ionization potential of CZT crystal ∼4.6 eV^32^, which leads to the conduction band edge at −3.03 eV and valance band gap edge −4.6 eV as shown in Fig. 1(b,c). The In-doping in CZT create shallow donor levels at 14 meV below the conduction band edge^33^, as shown in Fig. 1c.

**Figure 3:**
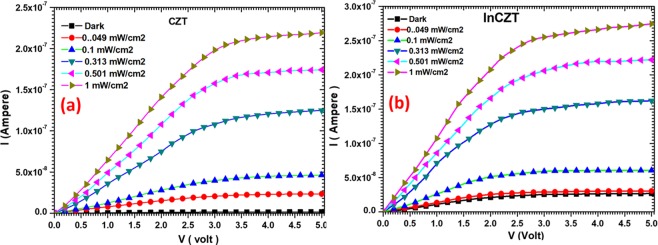
*I-V* characteristics of (**a**) CZT and (**b**) InCZT photodetector devices under different illumination power density.

Figure 3(a,b) shows the *I-V* characteristics of CZT and InCZT based photodetector devices under different power density of laser of 632.8 nm. It is observed from Fig. 3(a) that the dark current *I*_*d*_, for pure CZT photodetector is very small of the order of 1.38 nA, which is good for high-performance photodetectors^34,35^. On the other hand, In-doping provides additional free carriers which results in a higher dark current of 25 nA for InCZT photodetector. Under the illumination with laser light, electron-hole pairs generated, results in increase of device current as shown in Fig. 3(a,b). It is also visible from Fig. 3(a,b) that after reaching a specific applied bias the current achieved its saturation value and does not further increase with the increase of applied bias. This is because all the photogenerated carriers start moving for this applied bias and participate in photocurrent. There are no free carriers left to participate in current beyond this applied bias. The electrical parameters calculated from Hall effect measurements at room temperature found to be a mobility of μ_CZT_ = 1.57 × 10^2^ cm^2^/V.s and carrier concentration n_f_ = 1.72 × 10^11^cm^−3^ for CZT crystal. The InCZT crystal show slightly Improved carrier mobility of μ_InCZT_ = 2.34 × 10^2^ cm^2^/V.s might be due to slightly higher carrier concentration of n_f_ = 2.38 × 10^11^cm^−3^. Moreover, the carrier life time in CZT crystal was found to be 7.61 × 10^−2^ s while a longer life time was measured in InCZT of 9.83 × 10^−2^ s. The longer life time may be another reason for the improved carrier mobility in InCZT crystal.

**Figure 4:**
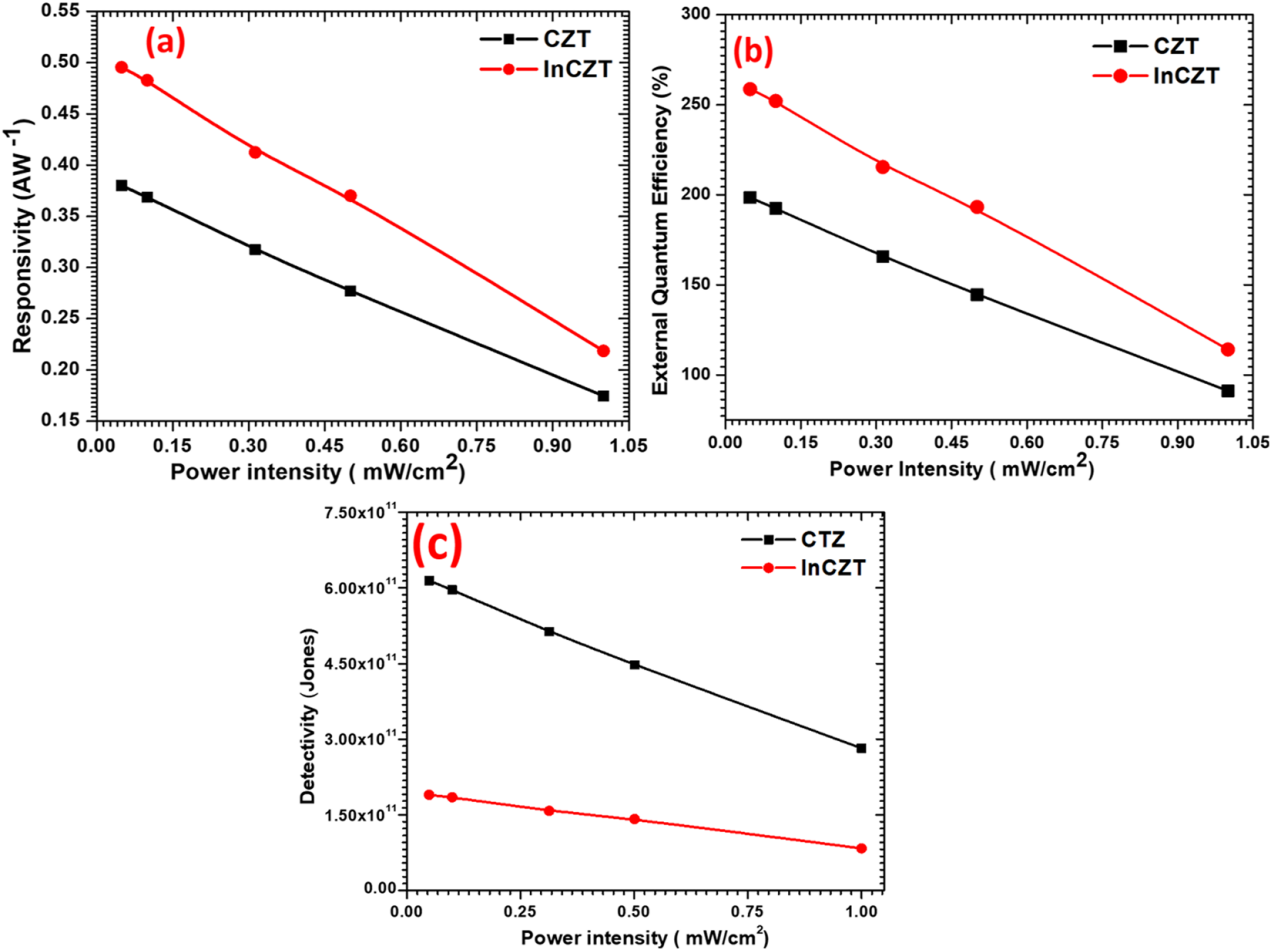
Illumination dependence (**a**) responsivity, (**b**) external quantum efficiency and (**c**) specific detectivity of fabricated CZT and InCZT photodetector measured under the applied bias of 5V.

Figure 4(a–c) shows the dependency of responsivity (R), external quantum efficiency and detectivity (D*) on incident illumination power density. These three parameters are very important for the evaluation of the sensitivity of photodetector. Responsivity is defined as the ratio of photocurrent generated $({I}_{p}={I}_{ph}-{I}_{dark})$ to the incident irradiation power (*P*_*in*_) on the effective area (A) of a photodetector^35–37^:

2 $$R=\frac{{I}_{p}}{{P}_{in}\times A}$$

As it is displayed by Fig. 4(a), the CZT based photodetector exhibits a responsivity of 0.37 AW^−1^ at illumination intensity of 0.049 mWcm^−2^ which decreases with the increase of illumination intensity. For the InCZT detector, the responsivity increase to 0.50 AW^−1^ for the same illumination and follow the identical pattern as CZT detector while remains higher responsivity for all illumination power density. The measured responsivity for our devices are larger than the previously reported values for PbI_2_ of 1 × 10^−4^ A W^−1^ ^34^, MoS_2_ of 7.5 × 10^−3^ A W^−1^ ^38^ and WS_2_ of 9.2 × 10^−5^ A W^−1^ ^39^.

The most important figures of merit for a photodetector is specific detectivity (D*), reflects the photodetectors sensitivity. Specific detectivity was calculated by relation^35–37^:

3 $${D}^{\ast }=R\sqrt{\frac{A}{2e{I}_{dark}}}$$

As shown in Fig. 4(b), the highest measured D* for pure CZT photodetector are calculated to be 6.00 × 10^11^ Jones at 5V bias. Doping of indium decreases the detectivity to 1.50 × 10^11^ Jones. As discussed above, the In-doping introduce a trap level below the conduction band in InCZT crystal. The presence of these trap states in the band gap of semiconductor act as the trapping and recombination center for the photogenerated charge carriers. Therefore, InCZT detector need slightly higher power to detect the input signal which results in lower detectivity as compare to pure CZT crystal.

The third figure of merit of a photodetector is EQE which was calculated by the equation^35–37,40^:

4 $$EQE=R\frac{hc}{e\lambda }$$

Where λ is illumination wavelength, c is speed of light, h is Planck constant and *e* is the electronic charge. It is observed by Fig. 4(c) that the highest calculated EQE of CZT based photodetector is 200% at 0.049 mWcm^−2^ illuminations and decreases with the increase of illumination intensity. For the InCZT based device, EQE increases to 250% and remains higher than the pure CZT based photodetector for all intensity. EQE also remains more than 100% for all illumination (except under the 1 mWcm^−2^ illumination of CZT photodetector). The measured EQE is higher than most of the previously reported devices such as PbI_2_ single crystal photodetector 49.6%^41^, MEH-PPV + CdS(Se) quantum dots of 60%^42^, CuPC/PTCBI of 75%^43^, PbS quantum dots + PCBM + P3HT of 51%^44^. The comparison of the key parameters of reported photodetectors with the present work are summarized in Table 1.

**Table 1:** Comparison of the performance of various photodetectors.

Photodetectors	Spectral range	Responsivity [AW^−1^]	Response times	Detectivity [Jones]	EQE (%)	Ref.
PbI_2_ nanosheets	450 nm	147.6	18 ms	2.56 × 10^11^	4.07 × 10^4^	^55^
PbI_2_ wire	450 nm	5.3	25 ms	1.6 × 10^10^	1476	
PbFI Nanosheets	365 nm	8	400 ms	5.8 × 10^11^		^25^
PbI_2_ nanosheets	530 nm	2.3	700 μs	1.5 × 10^12^		
PbI_2_ single crystal	450 nm	0.18	520 μs	3.23 × 10^11^	49.6	^41^
Graphene nanoribbons	1550 nm	1			80	^56^
Monolayer graphene	1470 nm	0.2				^57^
SnS_2_ nanosheets	850 nm	9.2 × 10^−4^			0.15	^58^
Multilayer Ta_2_NiSe_5_	808 nm	17.21			2645	^26^
MoS_2_	Visible	0.57	70 ms	≈10^10^		^59^
WS_2_	UV-Vis-NIR	0.70	9.9 × 10^3^	2.7 × 10^9^		^60^
CH_3_NH_3_PbI_3_ perovskite	550 nm	0.01	200 ms	2.60 × 10^10^		^46^
TlInSSe single crystal	532 nm	0.61 AW^−1^	300 ms	6.24 × 10^11^	120	^40^
CZT	633 nm	0.37	210 ms	6.00 × 10^11^	200	Present work
InCZT	633 nm	0.049	140 ms	1.50 × 10^11^	250	

**Figure 5:**
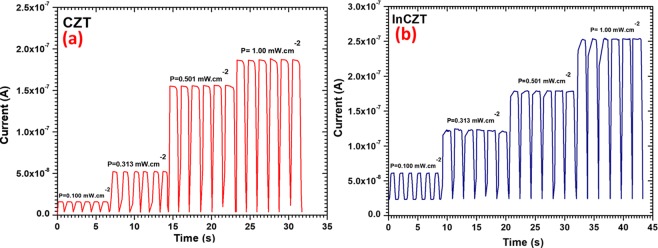
Photo-switching characteristics under different light illumination at 5V bias for (**a**) CZT and (**b**) InCZT.

**Figure 6:**
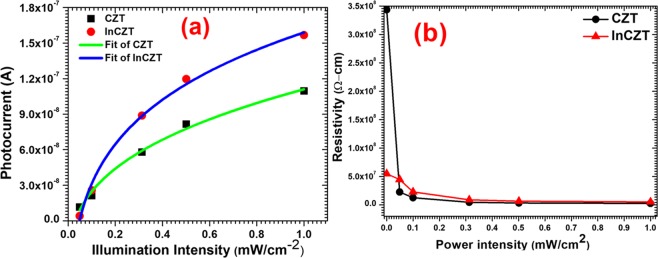
(**a**) Measured and fitted photocurrent and (**b**) resistivity of CZT and InCZT crystals under different light illuminations.

The switching behavior of pure and In-doped CZT photodetector devices are shown in Fig. 5(a,b), respectively. The time-dependent photocurrent of the photodetector was obtained by periodically turning ON/OFF the laser for different illumination intensity at 5V. When laser turned “ON” the current quickly increases to a saturation value and rapidly decreases when turned “OFF” and again attain the dark values (1.38 nA for CZT and 25 nA for InCZT). The corresponding rise and decay times are listed in Table 2. Rise time (*t*_*r*_) is defined as the time required to surge the photocurrent from 10% to 90% of saturation value while the decay time (*t*_*d*_) is the time required to fall its output from 90% to 10% of final output level^45^. It is observed from Table 2 that rise time for CZT based detector are in the range of 200–290 ms while decay time are in the range of 150 to 240 ms. For the InCZT based photodetector, the rise time decreases (in the range of 140–250 ms) and decay time increases (in the range of 200–280 ms). The decrease in rise time and increase in decay time for InCZT based detector can be understood in terms of decrease in resistivity or increase in conductivity of InCZT as compare to pure CZT [Fig. 6(b)]. Due to higher conductivity in InCZT, it is easy to start motion of charge carriers (decrease of rise time) when the laser is turned ON and difficult to stop the carriers when laser is turned OFF (an increase of decay time). In order to study the stability of the photodetectors, the laser was switched periodically at a constant interval for multiple cycles. The stability is good after five cycles which shows the robustness and reproducibility of the photodetector. As shown in Fig. 5, the ON/OFF switching behavior retained very well and reversible. The current in CZT photodetector rapidly increased from 1.38 nA to 209 nA as the laser turn on, giving a high photoswitch ratio (I_light_/I_dark_) of 152. The InCZT based photodetector showed a much lower photoswitch ratio ≈10 with a relatively high dark current of 25 nA.

**Table 2:** Rise and decay time of photodetector device based on pure CZT and In-doped CZT crystals for different illumination.

Illumination power density (mW/cm^2^)	Material	Rise time *t*_*r*_ (s)	Decay time *t*_*d*_ (s)
0.100	CZT	0.29	0.15
	InCZT	0.20	0.20
0.313	CZT	0.29	0.21
	InCZT	0.25	0.24
0.501	CZT	0.27	0.16
	InCZT	0.22	0.25
1.00	CZT	0.21	0.24
	InCZT	0.14	0.28

The different device performance of CZT and InCZT based photodetectors make it keen to further investigate their photocurrent generation mechanism. Various detection mechanisms have been reported, out of them photoconductive effect and photogating effect are the most commonly observed in photodetector devices^28,46,47^. The photoconductive effect involves a process in which photon absorption by a semiconductor generates excess free carriers and results in an increase in conductivity^47^. In contrast, photogating effect originates from long-lived charge trap states^26,48^. In this phenomenon, only one kind of the photogenerated carrier (electrons or holes) contributes to photocurrent while other kind of carriers get trapped in the localized states and induce extra gate voltage^49,50^.

**Figure 7:**
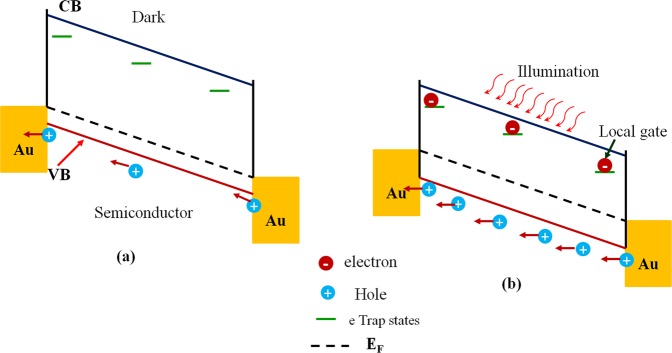
Photogating schematics. (**a**) Band alignment for a semiconductor channel with two Au contacts under external bias in dark (**b**) Band alignment under illumination.

In general, photoconductive effect involves the relationship that the photocurrent is linearly dependent on the illumination power density *ϕ*, described by ${I}_{P}\propto {\varphi }^{\beta }$ (β = 1), while photogating effect shows a sub-linear dependency (β < 1) on power density^21–25^. Therefore, we have plotted photocurrent as a function of illumination power density for both devices depicted in Fig. 6(a). The parameter of β is determined as 0.34 for CZT and 0.168 for InCZT, revealing the photogating effect is the dominant photocurrent generation mechanism in both photodetectors devices. The photogating effect can be explained by the schematic shown in Fig. 7. The low value of β attributed to long-lived charge traps due to higher defects density present in both semiconductors mainly in InCZT^6,12^. The CZT crystal has relatively higher number of native defects due to Cd and Te vacancies. Szeles *et al*. have reported that the Cd vacancies are the dominant native shallow acceptor defect with a concentration of ∼10^11^ cm^−3^ in CZT^51^. On the other hand, in addition to Cd-native and Te vacancies, In-doping create a shallow donor level below the conduction band in InCZT crystal as shown in Fig. 1b . These defect play an important role in the electrical compensation and carrier trapping in CZT which is the primary reason for photogating effect^52^. Figure 7(a) shows the energy band alignment of photodetector devices under external bias in the dark. Presence of traps in the band gap of semiconductor act as the trapping and recombination center for the photogenerated charge carriers. Under the illumination, the photogenerated electron gets trapped in these localized states near the conduction band (CB) edge and only photogenerated hole contribute to the photocurrent as shown in Fig. 7(b)^49,50,53,54^. The trapped electron act as a local gate and collectively generate a local electric field and therefore reduce the resistivity of devices under illumination as shown in Fig. 6(b). On the other hand, the photogenerated hole can recirculate many times during the lifetime of the trapped electron, leading to a high EQE (>100%) as depicted in Fig. 4(b). The higher value of β for CZT is due to better crystalline quality and a small defect in CZT compare to InCZT crystals^6,12^. The In-doping introduce additional defects in the crystal lattice of CZT and hence InCZT crystal have higher density of trapped electrons, this leads to a higher EQE of InCZT based devices as compared to CZT.

## Conclusions

In summary, we have successfully investigated photodetector performance of grown CZT and InCZT single crystals. The optoelectrical properties have been analyzed by measuring illuminance dependence *I-V* and *I-t* characteristics. The CZT photodetector exhibits a sensitive, fast and stable photoresponse to laser light of 633 nm. The In-doping in CZT crystal leads to deterioration of photoswitch ratio and specific detectivity while the improvement in responsivity and EQE of the device. Moreover, we have also investigated the photocurrent generation mechanism in both devices and found that the photogating effect is the dominant mechanism for both devices due to the presence of a large number of defects in the semiconductors. The preliminary results of our devices are superior to many other reported visible detectors, suggest that the CZT based crystal have desirable optoelectrical properties for future visible photodetector devices. The device performance can be further improved by reducing the defects, optimizing the growth parameters and using other dopant.

## Supplementary information

Supporting information
