# Supplementary material for: High-performance visible light photodetectors based on inorganic CZT and InCZT single crystals
Source: Sci Rep. 2019 Aug 27;9:12436. doi: 10.1038/s41598-019-48621-3 (PMC6711974; doi:10.1038/s41598-019-48621-3)
Supplement: Supplementary file 1 — Supporting information [file 41598_2019_48621_MOESM1_ESM.docx]

***High-performance visible light photodetectors based on inorganic CZT and InCZT single crystals***

***Mohd. Shkir^a^, Mohd Taukeer Khan^b^, I.M. Ashraf^c^, Abdullah Almohammedi^b^, E. Dieguez^d^, S. AlFaify^a^***

*^a^Advanced Functional Materials and Optoelectronics Laboratory (AFMOL), Department of Physics, College of Science, King Khalid University, Abha 61413, P.O. Box 9004, Saudi Arabia*

*^b^Department of Physics, Faculty of Science, Islamic University of Madinah, Madinah, Saudi Arabia*

*^c^Department of Physics, Faculty of Science, Aswan University, Aswan, Egypt*

*^d^Crystal Growth Lab, Departamento de Física de Materiales, Universidad Autónoma de Madrid, Madrid 28049, Spain*

Scheme-I
